# Available Evidence on the Diagnostic Accuracy of Chemiluminescence for Detecting Dysplasia or Malignant Transformation in Oral Potentially Malignant Disorders (OPMDs): A Systematic Review and Meta-Analysis

**DOI:** 10.3390/jcm15020815

**Published:** 2026-01-20

**Authors:** Fariba Esperouz, Mauro Lorusso, Giuseppe Troiano, Khristyna Zhurakivska, Domenico Ciavarella, Lorenzo Lo Muzio, Lucio Lo Russo

**Affiliations:** 1 Department of Clinical and Experimental Medicine, School of Dentistry, University of Foggia, 71121 Foggia, Italy; mauro.lorusso@unifg.it (M.L.); khristyna.zhurakivska@unifg.it (K.Z.); domenico.ciavarella@unifg.it (D.C.);; 2 Department of Medicine and Surgery, University LUM Giuseppe Degennaro, 70010 Casamassima, Italy; troiano@lum.it

**Keywords:** OPMDs, oral potentially malignant disorders, chemioluminescence, trasformation, early diagnosis

## Abstract

**Background**: Oral potentially malignant disorders (OPMDs) often exhibit heterogeneous clinical features, making the early detection of dysplasia very difficult. Several chemiluminescence-based devices, like ViziLite^®^, have been suggested as non-invasive adjuncts that can enhance the visualization of suspicious mucosal changes. However, their true diagnostic value remains unclear. **Methods**: A systematic review and meta-analysis were conducted in line with PRISMA 2020 guidelines. Thirteen clinical studies met the inclusion criteria, necessitating chemiluminescence as index test and histopathology as reference standard, with extractable 2 × 2 diagnostic data. For all OPMDs and leukoplakia-only subgroups, pooled sensitivity and specificity, DOR, SROC curves, and device-specific diagnostic accuracy were determined. **Results**: Of all the OPMDs, chemiluminescence demonstrated a high pooled sensitivity of 0.82 and a low specificity of 0.48 with considerable heterogeneity among studies. The results in the leukoplakia subgroup improved sensitivity of 0.87 and a specificity of 0.51 were recorded with a more concave SROC curve, which illustrated a better discriminative ability in keratinized lesions. Comparison of devices illustrates accuracy was best for ViziLite + Lugol iodine (~0.82) followed by standard ViziLite (~0.62) and ViziLite Plus (~0.53). **Conclusions**: Chemiluminescence, while it may demonstrate good sensitivity, has repeatedly shown to have limited specificity in a consistent manner, particularly in populations with mixed OPMD where inflammatory and benign lesions inflate the false-positive rates. Notably, diagnostic performance was higher in leukoplakia, suggesting that keratinized lesions benefit most from this adjunctive tool. Overall, chemiluminescence may facilitate lesion visualization and biopsy site selection but cannot supplant histopathological examination as a definitive diagnostic modality.

## 1. Introduction

Oral potentially malignant disorders (OPMDs) represent a heterogeneous group of mucosal alterations that include leukoplakia, erythroplakia, oral lichen planus, chronic hyperplastic candidiasis, oral submucous fibrosis, and epithelial dysplasia. These lesions have an increased risk of malignant transformation to OSCC [1]. The early detection of epithelial dysplasia within the lesion is one of the major challenges in oral medicine. Most OPMDs present with nonspecific, mild, or overlapping clinical manifestations [2]. Their appearance could vary from harmless-looking white or red spots to ulcerated or combined types that often mimic common benign inflammatory or reactive conditions [3]. Such clinical variability could lead to underestimation of the malignant potential and result in diagnostic delays [4].

Traditional visual examination, though widely used, may not be adequate for distinguishing high-risk lesions from low-risk alterations. Indeed, several studies reported that in clinically heterogeneous lesions, visual inspection may overlook subtle architectural changes or the most biologically active sites [5]. In this light, diagnostic uncertainty further extends to the determination of the most suitable biopsy site, even more so regarding large or multifocal OPMDs, where sampling error can occur and sometimes the taking of multiple biopsies is required, as has also been stated by several clinical studies, such as those related to the application of preoperative imaging modalities in OSCC [6].

To address these limitations, non-invasive adjunctive approaches are increasingly being investigated. Chemiluminescence has received a lot of interest due to its simplicity, speed, and affordability. ViziLite^®^, ViziLite Plus^®^, and MicroLux™ DL devices use a chemical process to generate high-intensity blue–white light, which is routinely rinsed with 1% acetic acid before use [7]. This combination creates an “aceto-white” effect, enhancing the reflectance of epithelial surfaces and the visibility of areas with altered optical properties [8]. Dysplastic epithelium, characterized by increased nuclear density, hyperkeratosis, architectural disorganization, and modified scattering coefficients, appears brighter under chemiluminescent light, thus potentially revealing preclinical conditions that may not be visible under traditional lighting conditions [9]. Despite a very good sensitivity of chemiluminescence for the identification of suspicious sites, its specificity is limited [10]. Inflammatory lesions, traumatic keratosis, and fungal infections can also present increased whiteness and hence lead to false-positive outcomes [3]. For this reason, chemiluminescence cannot be considered a diagnostic tool that can differentiate between benign and dysplastic tissue, nor can it replace biopsy, still representing the gold standard for epithelium examination [5].

In light of the growing interest in non-invasive technologies for the assessment of OPMDs, understanding the real diagnostic value, advantages, and limitations of chemiluminescence is of paramount importance. Therefore, the present study was designed to critically and systematically review the literature produced up to date regarding diagnostic accuracy of chemiluminescence and its possible role in clinical management of potentially malignant oral lesions.

## 2. Materials and Methods

This systematic review and meta-analysis were conducted in accordance with the Preferred Reporting Items for Systematic Reviews and Meta-Analyses (PRISMA 2020) guidelines (Prisma check list is provided in Supplemental Materials). The protocol for this review was prospectively registered in the International Prospective Register of Systematic Reviews (PROSPERO) under the registration number: CRD42024581512.

### 2.1. Search Strategy and Database Screening

A comprehensive literature search was conducted in PubMed, Scopus, and Web of Science (WOS). The search included all available records up to November 2025, with no restrictions on publication year. The PubMed search strategy combined MeSH terms and free-text words using Boolean operators as follows: “chemiluminescence AND (oral potentially malignant disorders OR OPMD)”.

Equivalent search strategies were adapted for Scopus and WOS and are provided in Supplemental Materials (Table S1). Additionally, the reference lists of relevant systematic reviews were manually screened to identify any further eligible studies.

### 2.2. Eligibility Criteria

Clinical studies evaluating the diagnostic performance of chemiluminescence-based devices (e.g., ViziLite, ViziLite Plus, ViziLite with Lugol/Toluidine Blue) in patients with oral potentially malignant disorders, including leukoplakia, were considered eligible. Studies were required to use histopathology as the reference standard and to provide sufficient information to reconstruct a 2 × 2 contingency table (TP, FP, FN, TN). Both prospective and retrospective full-text clinical studies published in English were included.

Studies were excluded if histopathology was not used as the reference standard, if diagnostic data were insufficient for 2 × 2 table reconstruction, or if they constituted non-clinical investigations (e.g., laboratory studies, reviews, case reports). Publications with overlapping datasets and studies assessing chemiluminescence for conditions other than OPMD were also excluded.

### 2.3. Focused PICO Question and Outcome Measures

(P) Participants: Patients with oral potentially malignant disorders (OPMD), undergoing diagnostic evaluation for dysplastic or malignant transformation.

(I) Intervention: Diagnostic assessment using chemiluminescence-based devices (e.g., ViziLite, ViziLite Plus, ViziLite with Toluidine Blue/Lugol).

(C) Comparison: Reference standard histopathological examination of biopsied lesions.

(O) Outcomes: Primary outcomes included: Sensitivity and specificity for detecting dysplasia or carcinoma; Diagnostic Odds Ratio (DOR); SROC curve performance.

### 2.4. Studies Screening and Inclusion

Two authors (FE and ML) independently screened all retrieved citations by evaluating titles and abstracts against the predefined inclusion and exclusion criteria. After this initial screening, potentially eligible studies were selected for full-text review to assess their suitability for inclusion in the qualitative and quantitative synthesis. In cases of disagreement or uncertainty, a third independent reviewer (LLR) was consulted to reach a final consensus.

### 2.5. Data Extraction

Data extraction was performed independently by two reviewers (FE,ML) using a standardized data collection form. Extracted variables included: first author and year of publication, country, study design, sample size, type of lesions, methods used for the diagnosis of OPMDs.

### 2.6. Assessment of Risk of Bias

The assessment of study quality and risk of bias was conducted using the QUADAS-2 [11], which evaluates the domains of selection, comparability, and outcomes for each included study. Studies rated as having critical or serious concerns were considered at high risk of bias. Two reviewers (Fe and ML) independently carried out the assessment, and any disagreements were resolved through discussion with a third reviewer (LLR).

The DeepL tool (https://www.deepl.com) was used to refine and rephrase certain sentences in the manuscript.

## 3. Results

### 3.1. Study Selection

**Figure 1:**
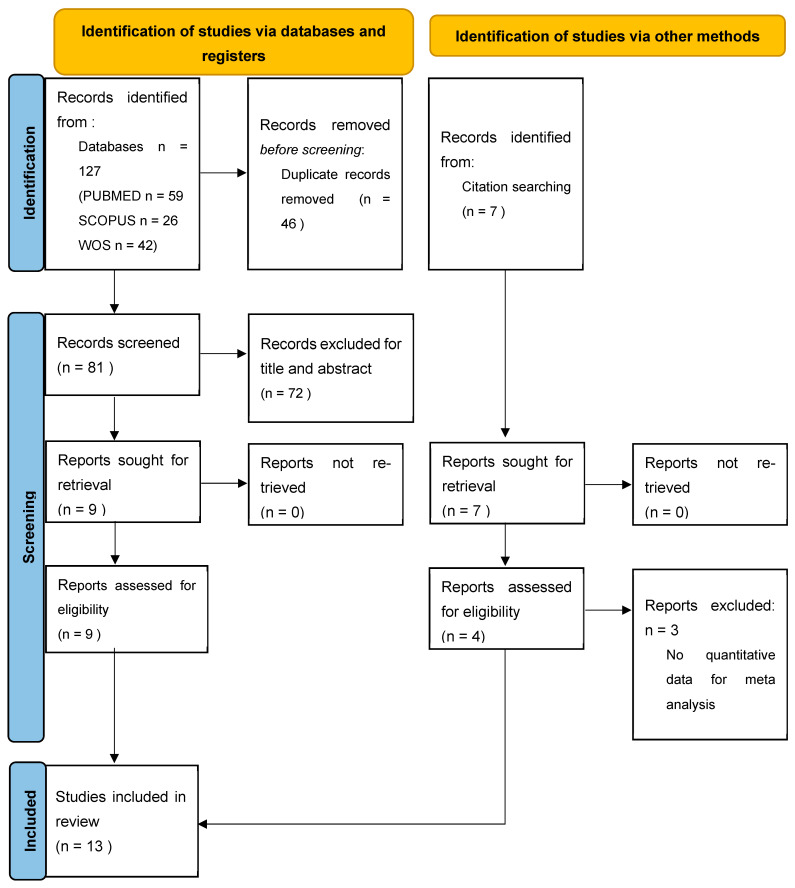
PRISMA 2020 flow diagram for new systematic reviews which included searches of databases, registers and other sources.

The electronic search retrieved a total of 127 articles from the electronic database search and 7 articles from the hand search published up to Novembre 2025. After the removal of duplicates (46), title and abstract analysis was performed on 88 articles, of which 72 were excluded after the screening of titles and abstracts. Full text analysis was performed on the remaining 16 articles; 3 of them were excluded because the absence of quantitative data analysis (Table S2: Excluded studies). The final review included 13 articles [12,13,14,15,16,17,18,19,20,21,22,23,24] (Figure 1).

### 3.2. Risk of Bias Assessment

Across the 13 included studies [12,13,14,15,16,17,18,19,20,21,22,23,24], the overall methodological quality was variable, with several recurrent limitations. Most studies demonstrated a high risk of bias in the patient selection domain [16,17,20,23]. Risk of bias for the index test was generally low across the included studies.

In nearly all cases, chemiluminescence (ViziLite or similar devices) was performed according to standardised manufacturer protocols and the examiners were usually unaware of the histopathological outcomes. The reference standard domain showed a low risk of bias in most studies that used histopathology to examine all lesions [14,16,21,23].

**Figure 2:**
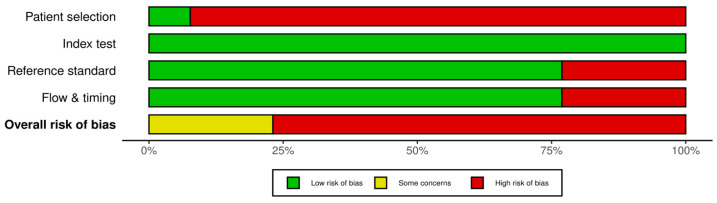
Risk of bias assessment of the 13 articles included in the review. Plot generated with robvis tool.

Several studies had issues in the ‘Flow & Timing’ domain, particularly those using selective biopsy strategies [15,17,19]. Across all studies, there were consistently high applicability concerns in the patient selection domain due to the exclusive inclusion of subjects who had already been referred to specialist clinics or were presenting with overtly suspicious lesions (Figure 2).

### 3.3. Study Characteristics

A total of 13 clinical studies evaluating chemiluminescence for the detection of oral potentially malignant disorders (OPMDs) were included in this review. The studies were conducted across a broad geographical distribution, including India [17,19,21,23], Pakistan [13,14], United Kingdom [12], Spain [24], Germany [16], Malaysia [20], Poland [18], and Australia [15]. Most studies adopted a cross-sectional or diagnostic accuracy design, with only a minority including prospective enrolment [20]. Sample sizes varied considerably, ranging from 30 to 126 patients, often reflecting the tertiary-care setting in which these investigations were conducted. Across the studies, a heterogeneous spectrum of oral mucosal lesions was evaluated. Some studies focused specifically on oral leukoplakia [13,14,16,21,23], whereas others included broader categories of OPMDs such as erythroplakia, oral submucous fibrosis, and lichen planus [17,18,19,20,21,22]. Several studies also included benign inflammatory lesions or normal mucosa controls for comparison [12,15,19].

All studies used chemiluminescence as the index diagnostic test, most commonly employing ViziLite or ViziLite Plus, sometimes in combination with complementary adjunctive methods such as toluidine blue staining [13,14,17,19,21] or Lugol’s iodine [23]. A few studies compared chemiluminescence with additional optical tools, such as VELscope [12,16,22], allowing partial assessment of multiple adjunctive aids.

In all studies, histopathology served as the reference standard for the confirmation of dysplasia or malignancy, although the extent of biopsy verification varied. Some studies biopsied all lesions regardless of clinical appearance [16,17,18,19,20], whereas others performed biopsies selectively based on clinical suspicion or test positivity [12,13,14,15,16,17] (Table 1).

## 4. Meta-Analysis

### 4.1. Chemiluminescence Specificity in OPMD

**Figure 3:**
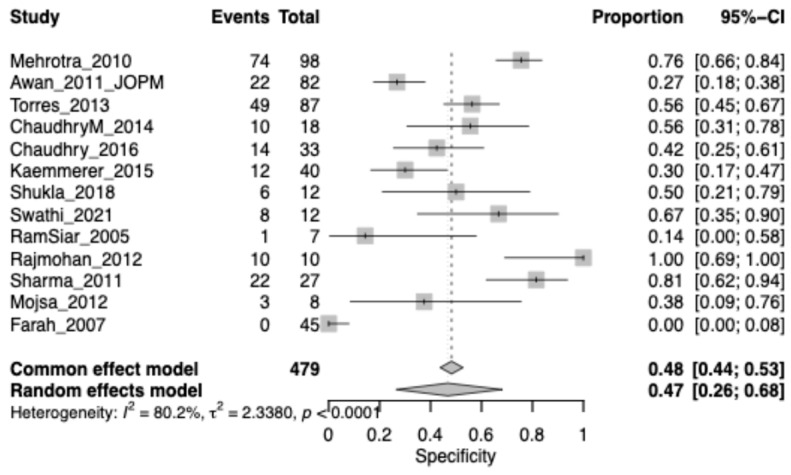
Forest plot showing pooled specificity of chemiluminescence for detecting OPMD lesions across included studies; Squares represent individual study estimates, with size proportional to study weight; horizontal lines indicate 95% confidence intervals. The diamond represents the pooled estimate.

The pooled specificity for OPMDs was 0.48 (95% CI: 0.44–0.53), highlighting a substantial rate of false positives when chemiluminescence is used alone. Heterogeneity was very high (I^2^ = 80.2%), reflecting large differences in diagnostic thresholds and lesion characteristics across studies (Figure 3).

### 4.2. Chemiluminescence Sensitivity in OPMD

**Figure 4:**
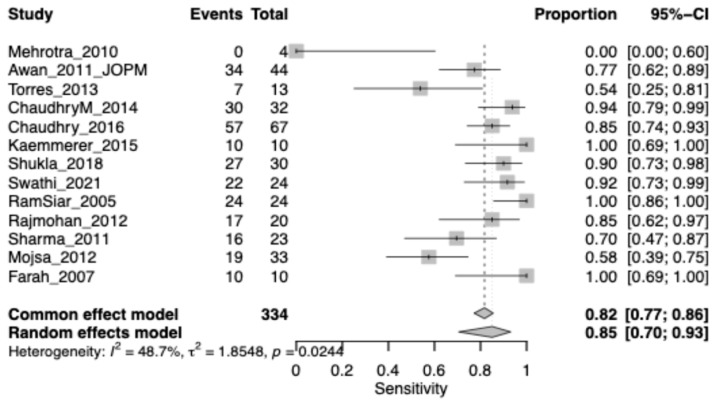
Forest plot showing pooled sensitivity of chemiluminescence for identifying OPMD lesions. Squares represent individual study estimates, with size proportional to study weight; horizontal lines indicate 95% confidence intervals. The diamond represents the pooled estimate.

The pooled sensitivity of chemiluminescence for detecting OPMDs was 0.82 (95% CI: 0.77–0.86), indicating a generally high ability to identify true positive lesions. Heterogeneity was moderate (I^2^ = 48.7%), suggesting some variability among studies, but not sufficient to question the overall reliability of the estimate (Figure 4).

### 4.3. Chemiluminescence Specificity in Leukoplakia Lesion

**Figure 5:**
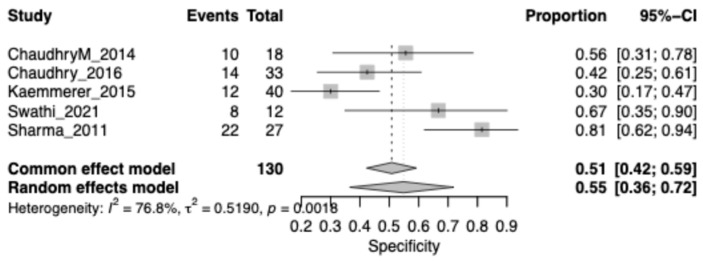
Forest plot showing pooled specificity of chemiluminescence restricted to leukoplakia lesions. Squares represent individual study estimates, with size proportional to study weight; horizontal lines indicate 95% confidence intervals. The diamond represents the pooled estimate.

The pooled specificity for Leukoplakia was 0.51 (95% CI: 0.42–0.59), highlighting a substantial rate of false positives when chemiluminescence is used alone. Heterogeneity was very high (I^2^ = 76.8%), reflecting large differences in diagnostic thresholds and lesion characteristics across studies. (Figure 5).

### 4.4. Chemiluminescence Sensitivity in Leukoplakia Lesion

**Figure 6:**
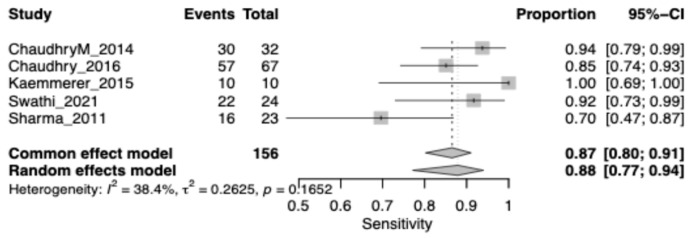
Forest plot showing pooled sensitivity of chemiluminescence restricted to leukoplakia lesions. Squares represent individual study estimates, with size proportional to study weight; horizontal lines indicate 95% confidence intervals. The diamond represents the pooled estimate.

The pooled sensitivity for Leukoplakia was 0.87 (95% CI: 0.80–0.91), highlighting a substantial rate of false positives when chemiluminescence is used alone. Heterogeneity was low (I^2^ = 38.4%) (Figure 6).

### 4.5. Accuracy of Chemiluminescence Devices

**Figure 7:**
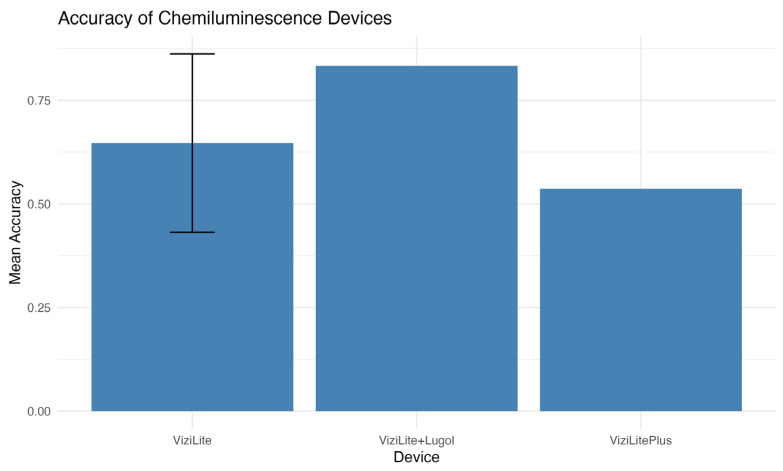
Accuracy of chemiluminescence devices.

The analysis shows that the ViziLite + Lugol device is the most accurate (~0.82), significantly outperforming the others. ViziLite has intermediate accuracy (around 0.62), but there is high variability between studies. ViziLite Plus is the least accurate (with an accuracy of ~0.53), but its performance is more stable (Figure 7).

## 5. Discussion

The current review and meta-analysis pooled data from thirteen clinical studies assessing the diagnostic accuracy of chemiluminescence devices for the detection of oral potentially malignant disorders. Across the included literature, some themes emerged. Most included clinical evaluations primarily consisted of patients with leukoplakia [13,14,16,21,23], although mixed groups of oral potentially malignant disorders, including erythroplakia, oral lichen plans, and submucous fibrosis, were also included [12,15,17,20,22,24]. Most clinical assessment designs included were standard cross-sectional, diagnostic accuracy studies conducted in either tertiary care clinics or oral medicine practice, with histopathology used as standard gold diagnostic assessment. ViziLite and ViziLite Plus chemiluminescence was widely used, sometimes with Lugol’s iodine and/or toluidine blue staining.

On analysing the diagnostic performance in all forms of OPMD globally, chemiluminescence was found to be moderately sensitive with poor specificity. This can be explained by the nature of all forms of OPMD, in which conditions such as lichen planus and inflammation/mucosal atrophy may result in increased reflectivity of light, thus high values of false positivity and considerable variability in specificity. Thus, analyses of mixed studies of all forms of OPMD revealed wide confidence intervals and considerable differences between individual studies in all pooled analyses. By contrast, analysis within the subtype of leukoplakia improved diagnostic accuracy considerably. Sensitivities tended to be higher and more concordant, with pooled figures reaching excellent diagnostic thresholds. Specificity, though remaining moderate, was appreciably better than in more mixed populations with OPMD. This can be explained by the biological nature of leukoplakia, characterized by its keratinized surface and generally even thickness of the epithelium, making it more effective at absorbing and reflecting light, and thus making subtle dysplasias more evident through chemiluminescence [25]. Taken together, all these points suggest that chemiluminescence is generally better suited to its application in keratinized lesions, but not so in mucosal pathologies, which may be more heterogeneous and/or include significant inflammatory aspects.

From a clinical perspective, the implications of false negative results deserve particular attention. While the sensitivity of chemiluminescence was relatively high, a false negative result cannot, therefore, be ruled out completely. The implications, therefore, of a false negative result for chemiluminescence might result in a delayed biopsy and subsequent diagnosis of epithelial dysplasia and malignant change. It must, therefore, not be used to rule out a disease process when there are chemiluminescence results that are negative. The analysis further investigated device-specific diagnostic accuracy. Though all studies included ViziLite-based systems, considerable variation was seen among devices. Accuracies tended to be moderately higher with ViziLite Plus than ViziLite, with mixed outcomes when combined with auxiliary agents such as toluidine blue or Lugol’s iodine. Such inconsistencies may be ascribed to variations in illumination strength, contrast, and criteria of assessment, including lesions included in varying analyses. Yet, inter-device variation in accuracy ranges indicates that chemiluminescence accuracy is not entirely dependent on devices but heavily reliant upon lesions.

Collectively, these results suggest that chemiluminescence can be considered an auxiliary method, but not a standalone one, in diagnosing oral lesions. In this context, chemiluminescence has to be put into perspective in a wider diagnostic setting involving other adjunctive and definitive diagnostic modalities. Chemiluminescence, oral cytology, and histopathological examination represent complementary and not competing diagnostic tools in the evaluation of OPMDs. While histopathology is considered as the gold standard for definite diagnosis and grading of epithelial dysplasia [26], its invasiveness and limited feasibility for repeated monitoring restrict its use as a first-line diagnostic modality. Chemiluminescence is a chairside, adjunctive technique that is noninvasive but only of relatively high sensitivity with consistently low specificity, as confirmed by the present meta-analysis. This profile makes chemiluminescence unsuitable as a diagnostic test on its own, but may be useful for lesion visualization and detection of clinically suspicious areas that needed to be investigated further. Oral cytology, in particular, when carried out using liquid-based methods and supported by molecular or immunocytochemical investigations, has reported relatively high sensitivity in the detection of epithelial dysplasia with variable but generally higher specificity compared with chemiluminescence [27]. Thus, this minimally invasive technique may represent an intermediate diagnostic step from clinical examination to biopsy, especially in large, multifocal, or clinically doubtful lesions.

In this context, compared with previous systematic reviews and meta-analyses [10,28] on chemiluminescence in oral potentially malignant disorders, the present study provides a more clinically oriented perspective. While earlier analyses primarily focused on screening performance or compared multiple light-based diagnostic modalities across heterogeneous disease entities, this work specifically restricted inclusion to OPMDs with histopathology as the reference standard and explored lesion-based differences in diagnostic accuracy. In light of these considerations, it is possible to propose a stepwise diagnostic approach: initial clinical examination in association with enhanced lesion detection and site selection guidance by chemiluminescence, oral cytology for risk stratification in selected cases, and confirmation and grading of dysplasia through definitive histopathological examination. An integrated diagnostic algorithm like this could achieve early detection of high-risk OPMDs while minimizing unnecessary biopsies and optimizing clinical decision-making.

### 5.1. Limitations

Limitations of the systematic review and meta-analysis conducted in this review include the following. Firstly, the number of studies available for the calculation of the sensitivity and specificity of chemiluminescence in the diagnosis of oral potentially malignant disorders, and also the number of participating subjects, was small. This is based on the available evidence. Secondly, the heterogeneity found among the studies included was substantial, and this could well be attributed to differences between the studies, including designs, types of the lesions, the chemical devices used, and the criteria employed to determine if the results were positive. Though the random-effects model was employed to treat this heterogeneity, the heterogeneity still cannot be ruled out. Third, some of the included studies used selective biopsy approaches according to suspicion or test positivity, which might have resulted in partial verification bias.

### 5.2. Clinical Implications and Future Directions

From a clinical viewpoint, chemiluminescence, in and of itself, is not primarily used as a diagnostic method but can be an effective supplementary diagnostic tool in certain instances. The high level of sensitivity in leukoplakia indicates that this technology can be used to better define lesions and determine those areas necessitating biopsies. On the other hand, its persisting low specificity, particularly in mixed groups of individuals with OPMD, indicates that chemiluminescence can over-estimate and, by extension, be unable to accurately distinguish between benign and dysplastic lesions. This can potentially raise the incidence of unnecessary biopsies if used exclusively as a diagnostic criterion. The application of chemiluminescence can be most safely made supplemental, specifically to better analyse initial lesions, particularly those which are keratinized, with definitive analysis remaining under histopathological assessment.

## 6. Conclusions

Chemiluminescence has shown good sensitivity but a persistently low specificity in the diagnosis of oral potentially malignant disorders. Diagnostic performance was significantly influenced by the lesion type: accuracy was considerably higher in leukoplakia, where keratinization accentuates optical contrast, while it decreases in heterogeneous OPMD groups due to frequent false positives caused by inflammatory or atrophic mucosa. There were minimal differences between devices; however, systems based on ViziLite tended to have slightly higher accuracy compared to other devices. In general, chemiluminescence should be regarded as a valuable adjunct to enhance the visualization of lesions and selection of biopsy sites, rather than as a diagnostic tool by itself, since histopathology is an essential modality for the final diagnosis.

## Figures and Tables

**Table 1:** Characteristics of included studies.

First Author (Year)	Country	Study Design	Sample Size	Type of Lesions Evaluated	Diagnostic Methods Used
Awan (2011)	UK	Cross-sectional	126 patients	Leukoplakia, erythroplakia, dysplasia	ViziLite, VELscope, Toluidine Blue, Conventional Exam
Chaudhry (2014)	Pakistan	Clinical comparative study	50 patients	Leukoplakia	ViziLite, Toluidine Blue
Chaudhry (2016)	Pakistan	Cross-sectional	100 patients	Leukoplakia, erythroplakia, OSMF	ViziLite, Toluidine Blue
Farah (2007)	Australia	Diagnostic accuracy study	50 lesions	Oral potentially malignant disorders	ViziLite, Conventional Exam
Kaemmerer (2015)	Germany	Clinical diagnostic accuracy study	63 lesions	Leukoplakia	ViziLite, VELscope, Toluidine Blue
Mehrotra (2010)	India	Cross-sectional clinical study	102 lesions (mixed population)	Leukoplakia, erythroplakia, OSMF, inflammatory lesions	ViziLite, Toluidine Blue, Conventional Oral Examination
Mojsa (2012)	Poland	Cross-sectional	41 lesions	Leukoplakia & PMDs	Chemiluminescence (ViziLite), Toluidine Blue
Rajmohan (2012)	India	Comparative diagnostic study	30 patients	Normal mucosa, precancer, cancer	ViziLite, Toluidine Blue, Exfoliative Cytology, Histology
Ram & Siar (2005)	Malaysia	Prospective clinical study	46 lesions	SCC, dysplasia, leukoplakia, benign lesions	ViziLite, Toluidine Blue, Histology
Sharma (2011)	India	Cross-sectional	50 lesions	Leukoplakia	ViziLite, Toluidine Blue
Shukla (2018)	India	Cross-sectional	42 patients	Mixed OPMD (leukoplakia, erythroplakia, OSMF)	ViziLite, VELscope, Toluidine Blue, Histology
Swathi (2021)	India	Cross-sectional	50 leukoplakia patients	Leukoplakia	ViziLite + Lugol Iodine, Toluidine Blue
Torres (2013)	Spain	Cross-sectional	100 patients	Oral potentially malignant disorders	ViziLite vs. conventional exam

## Data Availability

This study is a systematic review and meta-analysis based on previously published data. No new datasets were generated or analyzed, and data sharing is not applicable.

## Supplementary Materials

The following supporting information can be downloaded at: https://www.mdpi.com/article/10.3390/jcm15020815/s1, Table S1: search strategy; Table S2: Excluded studies; Figure S1: QUADAS-2. Ref. [29] is listed in the supplementary.

## Abbreviations

The following abbreviations are used in this manuscript:

Abbreviations	Meaning
OPMD(s)	Oral Potentially Malignant Disorder(s)
OSCC	Oral Squamous Cell Carcinoma
OSMF	Oral Submucous Fibrosis
PMD(s)	Potentially Malignant Disorder(s)
SCC	Squamous Cell Carcinoma
TP	True Positive
FP	False Positive
TN	True Negative
FN	False Negative
DOR	Diagnostic Odds Ratio
SROC	Summary Receiver Operating Characteristic
CI	Confidence Interval
I^2^	I-squared statistic (eterogeneità)
PRISMA	Preferred Reporting Items for Systematic Reviews and Meta-Analyses
PROSPERO	International Prospective Register of Systematic Reviews
QUADAS-2	Quality Assessment of Diagnostic Accuracy Studies Tool
VELscope	Visually Enhanced Lesion Scope
ViziLite^®^/ViziLite Plus^®^	Sistemi di chemiluminescenza
HEAL ITALIA	Health Extended Alliance for Innovative Therapies, Advanced Lab-research, and Integrated Approaches of Precision Medicine
WOS	Web of Science
