# Supplementary figures and images for: Available Evidence on the Diagnostic Accuracy of Chemiluminescence for Detecting Dysplasia or Malignant Transformation in Oral Potentially Malignant Disorders (OPMDs): A Systematic Review and Meta-Analysis
Source: J Clin Med. 2026 Jan 20;15(2):815. doi: 10.3390/jcm15020815 (PMC12842294; doi:10.3390/jcm15020815)

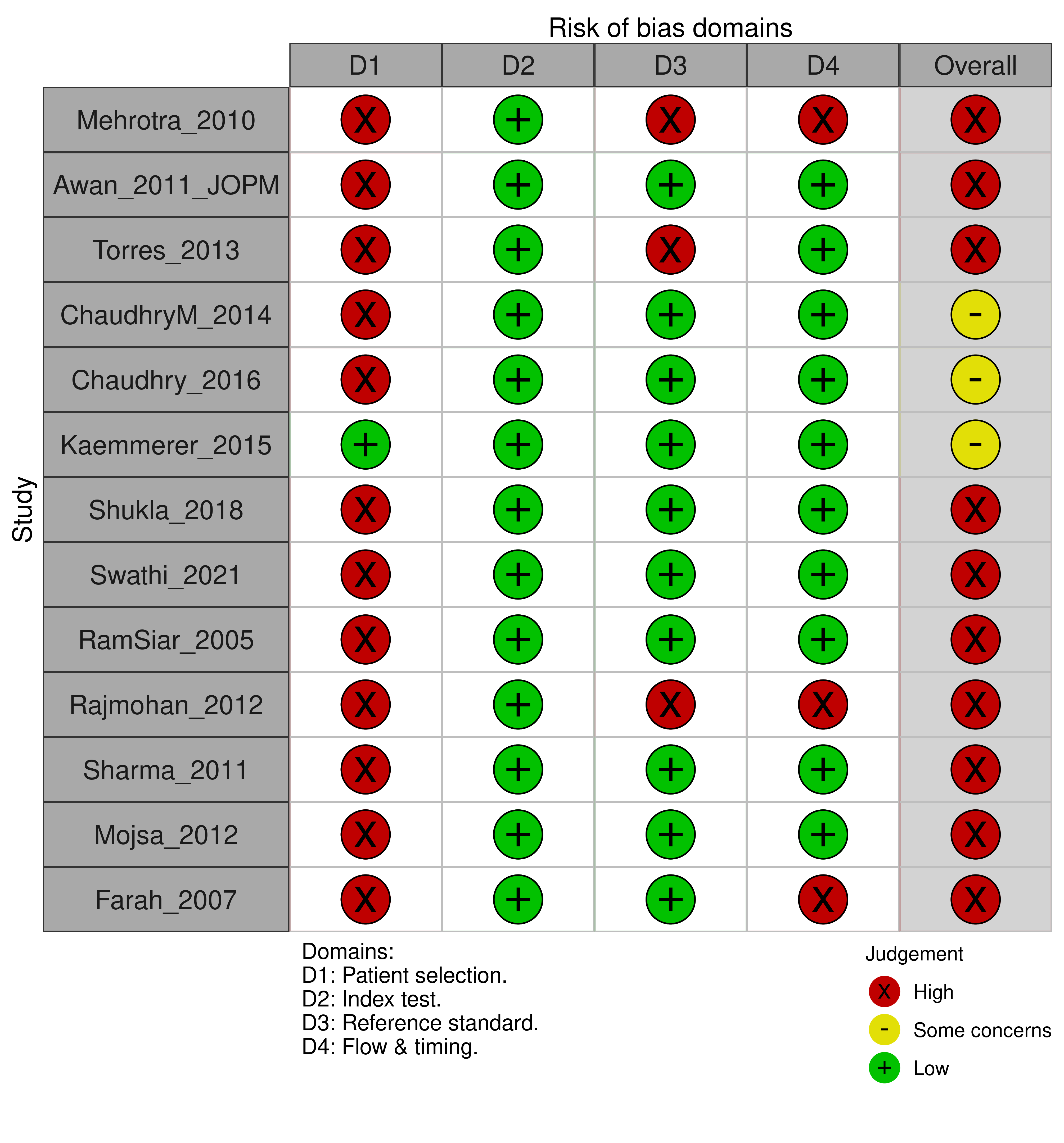

Supplement: Supplementary file 1 [file jcm-15-00815-s001.zip › QUADAS-2.png]
